# Bilirubin metabolism in relation to cancer

**DOI:** 10.3389/fonc.2025.1570288

**Published:** 2025-04-11

**Authors:** Fengyun Yi, Siyu Tao, Hongze Wu

**Affiliations:** ^1^ Department of Traditional Chinese Medicine, Jiujiang Hospital of Traditional Chinese Medicine, Jiujiang, Jiangxi, China; ^2^ The Second School of Clinical Medicine, Jiangxi Medical College, Nanchang University, Nanchang, Jiangxi, China

**Keywords:** bilirubin, tumor, metabolism, oxidation, inflammation, signal transduction, nanomedicine

## Abstract

Bilirubin, a metabolite of hemoglobin, was long thought to be a harmful waste product, but recent studies have found it to have antioxidant and anti-tumor effects. With the extensive research on the mechanism of malignant tumor development, the antioxidant effect of bilirubin is increasingly becoming a hotspot in anti-cancer research. At present, there are two main views on the relationship between bilirubin and cancer, namely, its pro-cancer and anti-cancer effects, and in recent years, studies on the relationship between bilirubin and cancer have not been systematically summarized, which is not conducive to the further investigation of the role of bilirubin on cancer. To understand the multifaceted role of bilirubin in tumorigenesis as well as to develop more effective and affordable antitumor therapies, this review provides an overview of the effects of bilirubin on tumors in terms of oxidative, inflammatory, and cellular signaling pathways, as well as the resulting therapeutic ideas and approaches.

## 1 Introduction

Cancer, an increasingly prevalent disease internationally, has become one of the principal causes of morbidity and death globally, with serious economic costs to society. As reported by the International Agency for Research on Cancer, the global cancer burden has risen, resulting in 19.3 million new cancer incidences and nearly 10 million deaths from cancer in 2020 alone (1). Although there are multiple treatment options for cancer, the outcomes remain unsatisfactory. Bilirubin, an endogenous antioxidant, is a widely used biomarker for diagnosing liver diseases. For a considerable time, bilirubin was regarded as a harmful metabolic waste. However, recent studies have revealed that there is a link between cancer and bilirubin levels. Mildly elevated bilirubin levels are generally related to a lower incidence of cancer and a better prognosis, but excessively high levels are linked to a higher incidence of cancer (2, 3). This article provides an overview of the diverse impacts of bilirubin metabolism on tumor progression and its utilization in cancer therapy, aiming to offer new ideas for improving tumor treatment.

## 2 Bilirubin metabolism process

**Figure 1:**
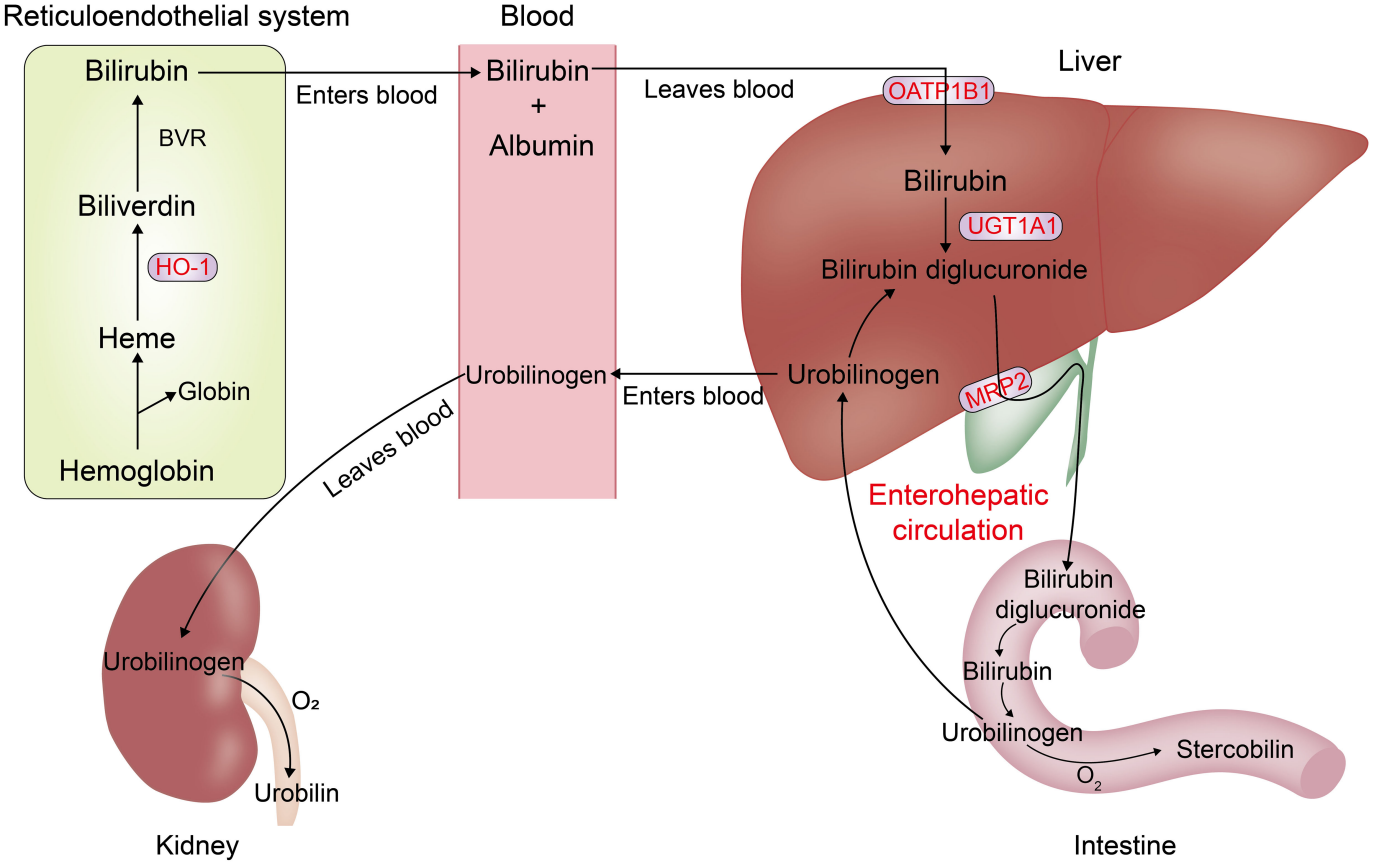
Metabolism of bilirubin in the body: Heme is decomposed by HO-1 (reticuloendothelial system) to produce bilirubin, which is then converted into indirect bilirubin by bilirubin reductase. Indirect bilirubin is absorbed by hepatocytes using UGT1A1, forming direct bilirubin, which is carried into the gallbladder, excreted via the bile ducts, transported to the intestines, and finally broken down by bacteria. Part of the products are recycled to enter the circulating pool of bilirubin (i.e., the enterohepatic circulation), and the rest is excreted through the kidneys and the intestines.

Bilirubin presents as a product of hemoglobin degradation and a bile pigment. Under physiological conditions, about three-quarters comes from senescent erythrocytes undergoing catabolism in the reticuloendothelial system, which contains splenic macrophages and hepatic Kupffer cells, and the remaining one-quarter of hemoglobin comes from ineffective red blood cell production and enzymes containing hemoglobin (4). In macrophages, heme is first decomposed to generate biliverdin by heme oxygenase-1 (HO-1), which then undergoes reduction to bilirubin by biliverdin reductase(BVR) (5, 6) (**Figure 1**). Bilirubin is a lipophilic molecule that accumulates in the cell, diffuses into the bloodstream, and then binds to circulating albumin to be transported to the liver. Unconjugated bilirubin (UCB, i.e., indirect bilirubin) is absorbed actively or passively by hepatocytes, mediated by the hepatic organic anion transporting polypeptides 1B1 (OATP1B1), and passes through uridine glucuronosyltransferase family1 memberA1 (UGT1A1) undergoes mono- or bi-glucuronidation (7) to form bilirubin monoglucuronide or bilirubin bi-glucuronide (i.e., direct bilirubin), which is excreted via the bile ducts by the multidrug resistance protein-2 (MRP2). It is first deposited in the gallbladder after the entry of direct bilirubin into the bile. Then it passes into the intestines along with many other bile components to promote the intake of fats and other fat-soluble chemicals. Bilirubin glucuronide is initially conjugated by microbial β-glucuronidase, and then the unconjugated bilirubin is oxidated and reduced by the intestinal flora (8). Several products can be reabsorbed back into the circulating bilirubin pool; while others are cast out through the intestines and kidneys, respectively, resulting in the characteristic colors of urine and feces.

## 3 Effects of bilirubin on the body

For a considerable time, bilirubin was regarded as a harmful metabolic waste. However, Serum bilirubin concentrations’ mild increases, such as those in patients with Gilbert’s syndrome (GS, a benign form of unconjugated hyperbilirubinemia), as well as levels in the upper quartile of the currently accepted physiologic serum bilirubin range, have been reported to be negatively correlated with a wide range of disorders within the human body.

It has been shown that bilirubin can down-regulate NAD(P)H oxidase to prevent diabetic nephropathy by being catabolized into biliverdin(BV), which has been demonstrated in rodents (9). Several studies have shown that mildly raised UCB also reduces the incidence of obesity, Alzheimer’s disease (10), metabolic syndrome (11, 12), nonalcoholic fatty liver disease (13, 14), and diabetes mellitus (15, 16). Thus, there is strong proof that bilirubin is a clinically important biomarker for reducing the prevalence of chronic diseases (16).

The positive effects of bilirubin on other chronic diseases have inspired scholars to study its relationship with cancer. It has been shown that mildly elevated bilirubin levels are generally related to a lower incidence of cancer and a better prognosis, but excessively high levels are linked to a higher incidence of cancer. Due to the lack of statistical studies related to bilirubin covering multiple cancers, the correlation between different cancers and bilirubin cannot be quantified at present and can only be roughly analyzed through existing independent studies. Several studies have shown that cancers with a strong association with bilirubin include liver, lung, and digestive tract cancers such as gastric (17), esophageal (18), and colorectal cancers, and gynecological cancers such as breast (19–21) and cervical (22) cancers. Since the liver, as an organ directly involved in many physiological processes including bilirubin metabolism processes, has a more complex relationship with bilirubin, the details of which will be described later, the sections other than hepatocellular carcinoma (HCC) are described here. As noted in a 2019 observational study, serum bilirubin has a strong negative correlation with all-cause mortality and is primarily motivated by the impact of bilirubin on cancer (2). In 2020, Horsfall et al. conducted a Mendelian randomization study that analyzed data from over 377,000 individuals from the UK Biobank. They found that people with the *rs887829* and *rs4149056* genes (genes that cause elevated levels of bilirubin) had a 17% reduced risk of suffering from lung cancer, a percentage that had an even larger value in the smoker population. This is because the extra bilirubin may help to counteract some of the effects of the reactive oxygen molecules that fill the lungs (23). The association of high bilirubin with low lung tumor risk has also been shown in other studies (22, 24–28). As another example, in several studies, a low prevalence of colorectal cancer was associated with high serum bilirubin concentrations or GS genotype presentation (29–31). Serum bilirubin levels were also found to be significantly lower in CRC patients than in controls in another study of colorectal cancer (32). However, Zhang and coworkers found in a retrospective study that high DBIL was strongly associated with a poorer postoperative prognosis in patients with stage II and stage III colorectal cancer. Subsequent studies confirmed these results (33, 34). This reflects the two-sided nature of bilirubin in cancer.

Genetic factors can influence cancer by affecting bilirubin levels, and in addition to *rs887829* and *rs4149056* described above, the Gilbert syndrome genotype (UGT1A1*28 purebred) (2) has been associated with elevated bilirubin levels. UGT1A1*28 allele carrier status is associated with a 20% reduction in CRC risk (32). Although bilirubin levels are highly heritable and genetic variation in *UGT1A1* explains a large portion of the variance, it has also been shown that enzymes involved in the production of bilirubin from hemoglobin, such as HO, may also have an effect (35). There is also the T(-413)T genotype of *rs2071746*, which has been shown to have significantly lower serum bilirubin levels in T(-413)T carriers in both the CRC group and the control group (32).

## 4 The significance of bilirubin in clinical practice and prognostic models derived from it

The above article describes the association of bilirubin with some cancers, which naturally leads to the question of whether bilirubin can be used as a way to optimize bilirubin-related cancer treatment and patient stratification, but it has been shown that, while individual serum markers are useful prognostic factors in the study of patients with cancer, individual markers may not be sufficient for predicting survival in the clinical setting. Combining multiple markers in a single index can improve their predictive ability (36–38). In addition to this, the performance of the single factor of bilirubin varies in studies targeting different cancers, e.g., in a study including male smokers as subjects, each 0.1 mg/dL decrease in bilirubin was associated with a 5% and 6% increase in the risk of lung cancer incidence and death, respectively (26), whereas an increase in bilirubin levels of 5 μmol/L in another study of smokers was associated with a lung cancer incidence rate decreased by 10.2/10,000 person-years (23). Therefore, this paper will only present the standard model related to bilirubin that has gained more acceptance. Several of these representative models will be described next.

### 4.1 total bilirubin

Total bilirubin (TB or TBIL) is one of the biomarkers reflecting the development of cancer and can be abnormal in diagnostic tests for cancers such as adenocarcinoma of the jugular and stomach. Total bilirubin can be used as a valid prognostic predictor for determining cancer prognosis, and valid prognostic evaluation tools can be developed based on total bilirubin. For example, researchers have developed a new prognostic score for adenocarcinoma of the jugular abdominal region according to the preoperative ratio of total bilirubin-albumin and fibrinogen-albumin by using a controlled study approach (39). Other researchers used a prospectively trained and retrospectively validated study methodology in 778 gastric cancer patients and analyzed using X-tile software, determined that the serum levels of TBIL and albumin were independent OS forecasters in patients of gastric cancer (17), and then low serum TBIL was linked to advanced gastric cancer and worse prognosis. Another study demonstrates a strong connection between high-level DBIL and poor postoperative prognostic results in stage II and stage III colorectal cancer patients (40). A multicenter study of the association between serum TB and mortality of all causes in cancer patients with cachexia also showed that patients with high TBIL levels had worse OS in the presence of serum total bilirubin greater than the normal range of between 1.7-17.1 μmol/L (≥21.7 μmol/L) (41). The prediction of cancer prognosis by TBIL has shown different results in different studies, which requires further research to explore its internal logic.

### 4.2 ALBI grade

The ALBI grade is a serviceable tool proposed by Philip J et al. in 2015 for assessing cancer status, as they ascertained objective measures of hepatic function (albumin and bilirubin) that independently affect the survival of HCC patients by utilizing data from extensive global databanks, assembling those to form a model comparable with the traditional C-P classification (42), which has a linear prediction equation of Linear prediction = (log10 bilirubin × 0.66)(albumin × -0.085), bilirubin in μmol/L and albumin in g/L. This model calculates patient-level linear predictions (xb) as well as applies cutpoints to assign every patient to any of the three prognosis divisions, now labeled ALBI classification, grades 1 to 3. Points of cut are xb ≤ -2.60 (ALBI class 1), > -2.60 ~ ≤ -1.39 (ALBI class 2), xb > -1.39 (ALBI class 3). Earlier studies have demonstrated that ALBI can help determine the prognosis of cancers such as HCC. In a study published in 2024, researchers found that ALBI and platelet-albumin-bilirubin(PALBI) grade were related to the prognosis of small cell lung cancer(SCLC) and could be utilized as simple, affordable, and a useful marker for the determination of follow-up therapies and prognosis of SCLC patients (43) by examining the association between ALBI grade and the PALBI grade and prognosis in SCLC patients, and as the grade scale increased, the mortality rate of the patients improves. Several studies published between 2021 and 2024 for HCC have shown that the ALBI grade plays a role in aiding outcome evaluation and liver reserve evaluation at several stages in stereotactic body radiotherapy of the liver, and thus it is helpful in determining the prognosis of HCC patients who undergo this therapy (44–46), and that the rate of hepatotoxicity occurs at an elevated rate as the grade increases. In addition, ALBI has been used in the assessment of the prognosis of various cancers such as squamous cell cancer of the esophagus (47) and SCLC (43). In summary, ALBI can be used as one of the factors for evaluating the prognosis of a wide range of cancers and can be a useful tool for determining prognosis as well as helping to make decisions related to cancer treatment.

Although the ALBI grade is very objective, its calculation process is quite complex. So Kariyama et al. introduced the easy-albumin-bilirubin grade (easy-albumin-bilirubin) in 2020, which is much easier to calculate in estimating hepatic functional reserve and is strongly linked with the original ALBI grade (48). A study published in 2024 in HCC has confirmed that the simple albumin-bilirubin grade is an objective and feasible prognostic model for assessing the abnormalities of liver function in HCC patients and is independent of the patient’s performance status.

### 4.3 PALBI grade

Platelets play a marker role during the course of portal hypertension in cirrhosis and may reflect HCC. Thus Roayaie et al. proposed the PALBI class at the liver meeting in 2015. The PALBI classification includes bilirubin, albumin, and platelet count in serum levels to signify liver reserve in HCC. When this score rises, it usually signals a poor prognosis for the cancer. The score has been validated in the prognostic assessment of cancers such as small-cell lung cancer (43) and HCC (49–51), and it has even been pointed out that in the model of platelet-albumin (PAL), the easy (EZ)-ALBI grade and the ALBI grade, PALBI, and end-stage liver disease (MELD), which are the several hepatic reserve models, PALBI was the best model (52). In summary, PALBI is an available prognostic assessment model in HCC as well as other cancers.

## 5 The effects and their mechanism of bilirubin on tumors

### 5.1 Tumor inhibitory effects of bilirubin

#### 5.1.1 Bilirubin inhibits oxidative stress to suppress tumor development

**Figure 2:**
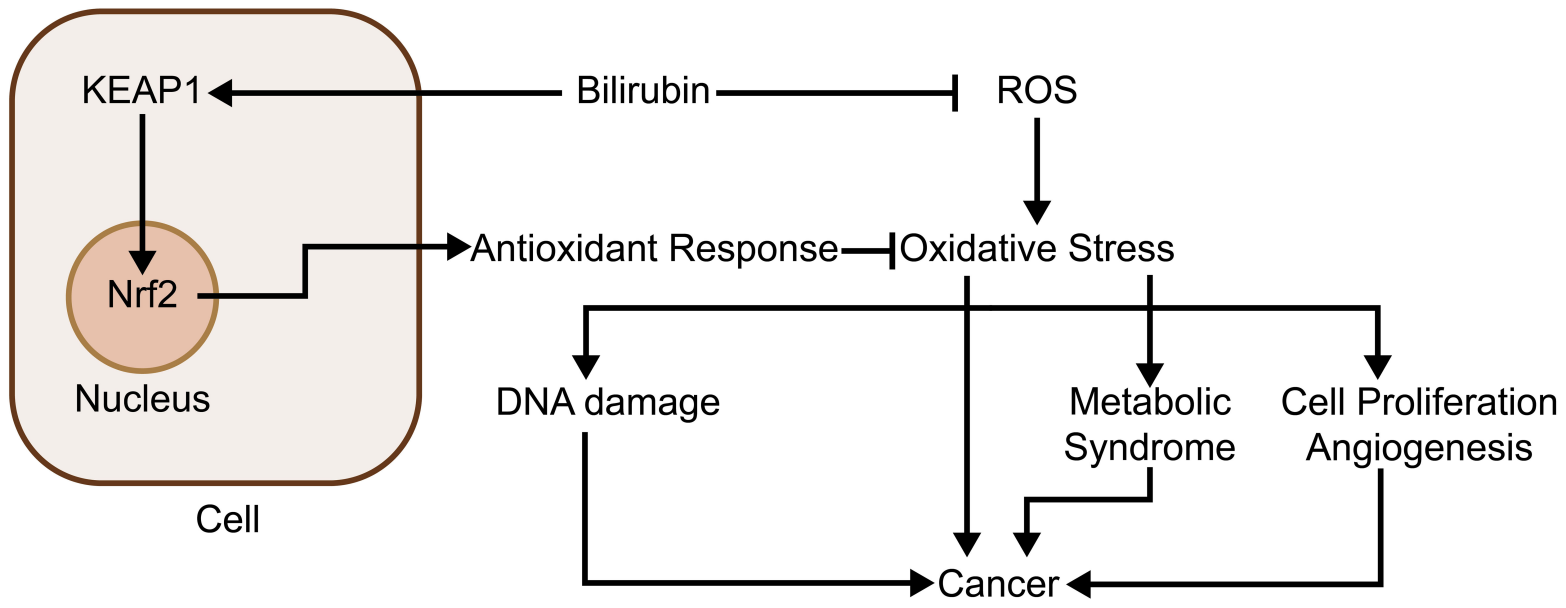
Bilirubin inhibits oxidative stress and thus tumor development: Bilirubin inhibits oxidative stress through two main pathways. First, it acts directly as an antioxidant to scavenge oxygen radicals, thereby inhibiting the high level of oxidative stress of cancer cells and selectively causing cancer cell apoptosis; second, it promotes the expression of several antioxidant genes by covalently binding to KEAP1 and activating the Nrf2 pathway. Through these two pathways, bilirubin also inhibits oxidative stress-induced diseases that are highly associated with cancer, such as metabolic syndrome.

In normal physiological circumstances, the presence of antioxidants counteracts the reactive oxygen species (ROS), resulting in a balance (53), while oxidative stress is a situation when the balance is disrupted. Cancer development and progression are closely associated with the processes of intracellular oxidative stress. The ROS overproduction results in oxidative impairment of lipids, proteins, and DNA (53), where the accumulation of DNA damage induced by ROS may lead to genetic destabilization in the case of cancer, thus facilitating the development of cancer (54). In addition to this, oxidative stress acts as a second messenger to promote cell proliferation (55) and angiogenesis (56), creating favorable conditions for tumor growth and development. Bilirubin is a potent free radical scavenger with antioxidant properties and reduces ROS, especially in lipid peroxidation (57, 58). In 1987, Stocker et al. proved in a study that bilirubin possesses characteristics of a natural ROS scavenger with superior antioxidant properties compared to those of vitamins C and E (59). Another study showed that every 1 mol of bilirubin scavenges 2 mol of oxygen free radicals, which in turn prevents excessive ROS from causing damage to the body (60). In addition to direct antioxidant responses, it has also been shown that bilirubin can indirectly function to the alleviation of oxidative stress, examples including activation of the nuclear factor erythroid 2-related factor 2 (Nrf2) pathway through covalently binding to Kelch-like ECH-associated protein 1 (KEAP1) (54, 61) and thus acting as an antioxidant in the cell. Thus bilirubin’s ability to scavenge ROS gives it an inhibitory function on cancer development and cancer-related inflammation. Bilirubin may also inhibit other diseases through its inhibitory effect on oxidative stress and thus inhibit tumors, for example, the occurrence of metabolic syndrome is closely related to oxidative stress (62), and some studies have also shown a negative relationship between the level of human serum total bilirubin and the incidence of metabolic syndrome (63–65), which in turn is positively correlated with the occurrence of many cancers, e.g., cancers of lung and colorectal (66–68), so bilirubin may inhibit the development of related cancers in this way. Besides that, in cancers that have already developed, bilirubin may also function to inhibit tumor progression while preserving normal cells. In studies on a variety of cancers, cancer cells have been shown to be under high levels of oxidative stress (69–75). In a model proposed in the literature, a decrease in the level of oxidative stress may contribute to the apoptosis of malignant cells and may serve as one of the mechanisms by which bilirubin screens malignant cells (76) (**Figure 2**).

#### 5.1.2 Tumor suppression by bilirubin effects on proto-oncogene/oncogene signaling pathways

##### 5.1.2.1 B-cell lymphoma-2

The Bcl-2 protein family supervises the integrity of the cellular genome to inhibit tumor growth (77). According to their function and structure, the proteins can be classified into three categories (78). The first is the Bcl-2 subfamily, which possesses a characteristic of apoptosis resistance (79). Then there is the Bcl-2-associated X protein(Bax) subfamily, which has pro-apoptotic activity and can form holes within the mitochondrial membrane (80), resulting in mitochondrial outer membrane permeabilization (MOMP) as well as a short discharge of cytochrome c (81, 82). The third group, the BH3 domain-containing proteins subfamily, is considered to be a lethal structural domain essential for apoptosis and critical for pro-apoptotic activity. UCB causes mitochondrial ROS production, which consequently causes activating *p38* and *p53* downstream, ultimately leading to the increased regulation of the pro-apoptotic protein Bax and the decreased regulation of the anti-apoptotic protein Bcl-2 and phosphorylation of Bcl-xL/Bcl-2 associated death promoter (Bad) (83), thus causing apoptosis. The same results can be found in another study for breast cancer (84). It follows that bilirubin may induce apoptosis in tumor cells by upregulating Bax and downregulating Bcl-2.

Bilirubin, an endogenous antioxidant, has a dramatic antitumor impact on the carcinoma of colon and rectum cell lines. It influences cell survival and tumor cell progress, possibly by regulating the expression level of the p53 protein, and consequently controls apoptosis and autophagy. Autophagy being a complex and environmentally relevant event, has been described to be associated with the occurrence of the carcinoma of colon and rectum. Autophagy confines the proliferation of cancer cells in the early stages and promotes cancer progression under stressful situations in the later stages. In one study, researchers found that bilirubin significantly inhibited autophagy in the human colon adenocarcinoma cell lines of LS180 and SW480 cells by suppressing Beclin-1 and Microtubule-Associated Protein 1 Light Chain 3B (LC-3B) in the cells, and increased apoptosis by the upregulation of *p53* in LS180 cells and downregulation of the *Bcl-2* gene which inhibits apoptosis in SW480 cells (85). However, the study of bilirubin’s influence on cancer development through autophagy is still incomplete, and the above study did not address the impact of autophagy in the progression of cancer, which still needs to be demonstrated in subsequent studies.

##### 5.1.2.2 NF-κB

Nuclear factor-κB (NF-κB) is a nuclear factor family consisting of multiple transcription factors involved in regulating inflammatory response, cell proliferation, tumor development, and invasion. Studies have shown that bilirubin and its metabolite biliverdin can inhibit this transcription factor.

A substantial quantity of experimental evidence demonstrates that NF-κB participates in the epithelial-mesenchymal transition (EMT) process vital for the local and distant progression of cancer by up-expressing the marker N-cadherin of mesenchymal and down-expressing the marker E-cadherin of epithelium (86). In addition, matrix metalloproteinases (MMPs), as the target of NF-κB action, play critical parts in physiological processes, e.g., organ growth and tissue metastasis (87). Studies in cervical cancer cells have found that NF-κB induces Epithelial-mesenchymal transition and stem cell-like characteristics of the tumor and promotes the tumor cells, self-renewal and migration (88). In cervical cancer cells, the modifications after translations of NF-κB were also reported to regulate tumor cell metastasis and invasion. The o-linked β-n-acetylglucosamine (o-glcnacylation) modification, dramatically elevated in cervical cancer cells, is the only intracellular glycan modification engaged in signaling. O-glcnacylation enhances the translocation of NF-κB by inhibiting the interaction of NF-κB with IκB. This enhanced the C-X-C chemokine receptor 4 (CXCR4) expression downstream, upregulated the expression of *HPV E6/E7*, and *Ki-67*, and then promoted the metastasis of uterine cervical cancer cells to the lung (89).

It is confirmed in mouse myocardial microvascular endothelial cell line H5V that UCB may influence the regulatory pathway of NF-κB via interaction with IKK proteins by inhibiting tnfα-stimulated NF-κB nuclear translocation (90). Another study also showed that astrocyte cultures showed a dramatic increase in tumor necrosis factor receptor1(TNFR1) levels, as well as subsequent activation of MAPKs p38, Jun N-terminal kinase1/2, and NF-κB, and signal-regulated kinase1/2, which is outside of the cell, when stimulated with UCB (91). The results suggest that bilirubin exerts a protective effect in intrinsic immune-related inflammation at certain doses by a mechanism associated with the NF-κB signaling pathway inhibition and the NOD-like receptor family CARD domain-containing protein 4 (NLRC4) activation, Absent in Melanoma 2 (AIM2), and NOD-like receptor family pyrin domain containing 3 (NLRP3) inflammatory vesicles, of which the inhibition contributes to the control of cancer (92–94).

Studies have shown that the metabolite of bilirubin, biliverdin, suppresses NF-κB activation caused by TNF-α, and overexpression of NF-κB stimulated by hbvr causes the cell cycle to halt in phase 1/0. This supports BVR and its substrates to regulate NF-κB, thus biliverdin can be used as a potential therapeutic tool to regulate NF-κB and is expected to inhibit the proliferation of tumor cells through this pathway (95). Biliverdin may affect NF-κB through at least two pathways. Biliverdin may inhibit NF-κB function by directly binding (96, 97). Biliverdin may also interfere with NF-κB activation signaling through its tetrapyrrole molecular structural properties (98). It has also been shown that bilirubin treatment reduced p-p65, p-IκBα, and IκBα protein levels to normal levels, suggesting that bilirubin specifically reduces NF-κB pathway activation associated with inflammation. Therefore, besides its potent anti-oxidative stress effects, bilirubin may also attenuate inflammation in osteoarthritis (OA) by inhibiting the NF-κB pathway (99), and some studies have suggested that osteoarthritis may promote the development of cancer, so bilirubin may serve to decrease the cancer development risk in this way (100).

##### 5.1.2.3 Ras/Raf/MEK/ERK

The Ras-Raf-MEK-ERK pathway integrates signals coming from the cell surface receptors into signaling pathways downstream that promote cell growth processes and proliferation in many categories of cells.

Bilirubin exerts an inhibitory effect on multiple sites of the RAS/RAF/MEK/ERK signaling pathway. For example, *in vitro* bilirubin damages the Raf/ERK/MAPK pathway activation and intracellular levels of Raf and cyclin D1, leading to hypophosphorylation of amino acids S608 and S780 by retinoblastoma proteins, which prevents the release of Yin Yang 1(YY1) into the nucleus and hinders the capability of YY1 to regulate gene expression and support cell proliferation. Bilirubin was also found to promote growth arrest in the cultures of human vascular smooth muscle primary cells stimulated by serum. Researchers have proposed that this is a result of the interaction of bilirubin with the Raf/ERK/MAPK pathway, its impact on the content of cyclin D1 and Raf, altering the hypophosphorylation profile of retinoblastoma protein, calcium efflux, and YY1 protein hydrolysis (101).

As for vascular growth, which is highly correlated with tumor development, bilirubin inhibits ERK activity in damaged blood vessels, and because ERK activation is associated with the mitogenic stimulation-induced cyclin D1 expression (102, 103), the reduction of ERK activity caused by bilirubin may be responsible for the down-regulation of cyclinD1 by bilirubin after arterial injury. Furthermore, the expression of the transcription factor p53 and the cyclin-dependent kinase inhibitor p21 induced by bilirubin inhibits the proliferation of established smooth muscle cells (SMC) and neoplastic endothelial proliferation (104, 105). The anti-proliferative effects of bilirubin may involve various mechanisms. In vascular SMC cultured *in vitro*, bilirubin reduces the activity of ERK (101), which is a key kinase for entry into the S phase. In addition, the reduction of ERK activation by bilirubin correlates with the ability of bilirubin to block airway SMC growth. However, during the mediation of the anti-proliferative effects of bilirubin on vascular SMC, the transcription factor p53 appears to act importantly. In injured blood vessels, researchers found bilirubin to be a powerful inducer of p53 expression. Furthermore, bilirubin facilitates p53 expression in a variety of cell types, and p53 deletion eliminates the anti-proliferative influence of bilirubin in mouse SMC. What is interesting is that p53 may also promote the ability of bilirubin to cause apoptosis in SMC cultured under serum-free or serum-restricted conditions due to its stimulation of the apoptosis signaling pathway (106). Besides, as p21 overexpression in vascular SMC has been demonstrated to restrain their migration, p21 may as well be involved in the anti-migratory effects of bilirubin (107). Furthermore, bilirubin inhibits nasopharyngeal cancer cell invasion by decreasing intracellular ROS levels and inhibiting ERK1/2 activation and MMP-2 expression (108). While many associations exist between bilirubin and blood vessels and smooth muscle, studies addressing bilirubin’s effect on tumor vasculature and smooth muscle tumors are scarce and require further study.

##### 5.1.2.4 PKA

**Figure 3:**
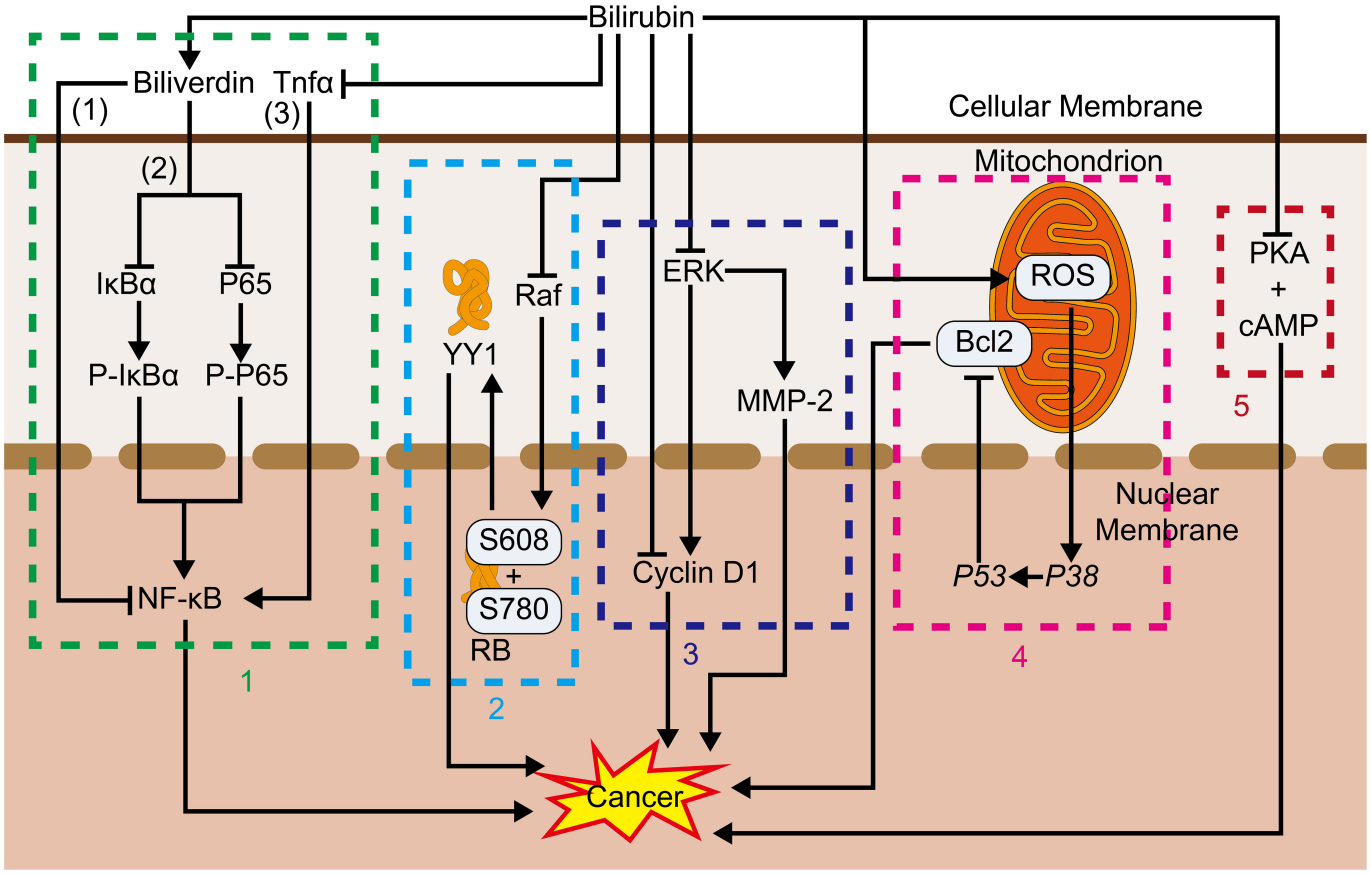
1. NF-κB: (1) Bilirubin promotes tumor development by altering immune cell phenotype. (2) Bilirubin, converted from bilirubin, also inhibits IκBα and P65 associated with NF-κB activity thereby inhibiting NF-κB. (3) Bilirubin also inhibits Tnfα associated with NF-κB activation thereby inhibiting its function. 2. Raf, Bilirubin inhibits Raf, and thus retinoblastoma protein, which in turn inhibits the release of YY1 into the nucleus and blocks its proliferation-promoting process. 3. ERK, Bilirubin inhibits proliferation and cancer metastasis by inhibiting ERK, thereby inhibiting cyclin D1 and MMP-2; Bilirubin can also inhibit proliferation by directly inhibiting cyclin D1. 4. Bcl2, Bilirubin promotes the production of ROS in mitochondria, which activates *P38*, and its activation of *P53* inhibits *Bcl2*, which in turn inhibits the anti-apoptotic process. 5. PKA, Bilirubin inhibits the binding of PKA to cAMP thereby inhibiting its pro-value-added effects.

**Figure 4:**
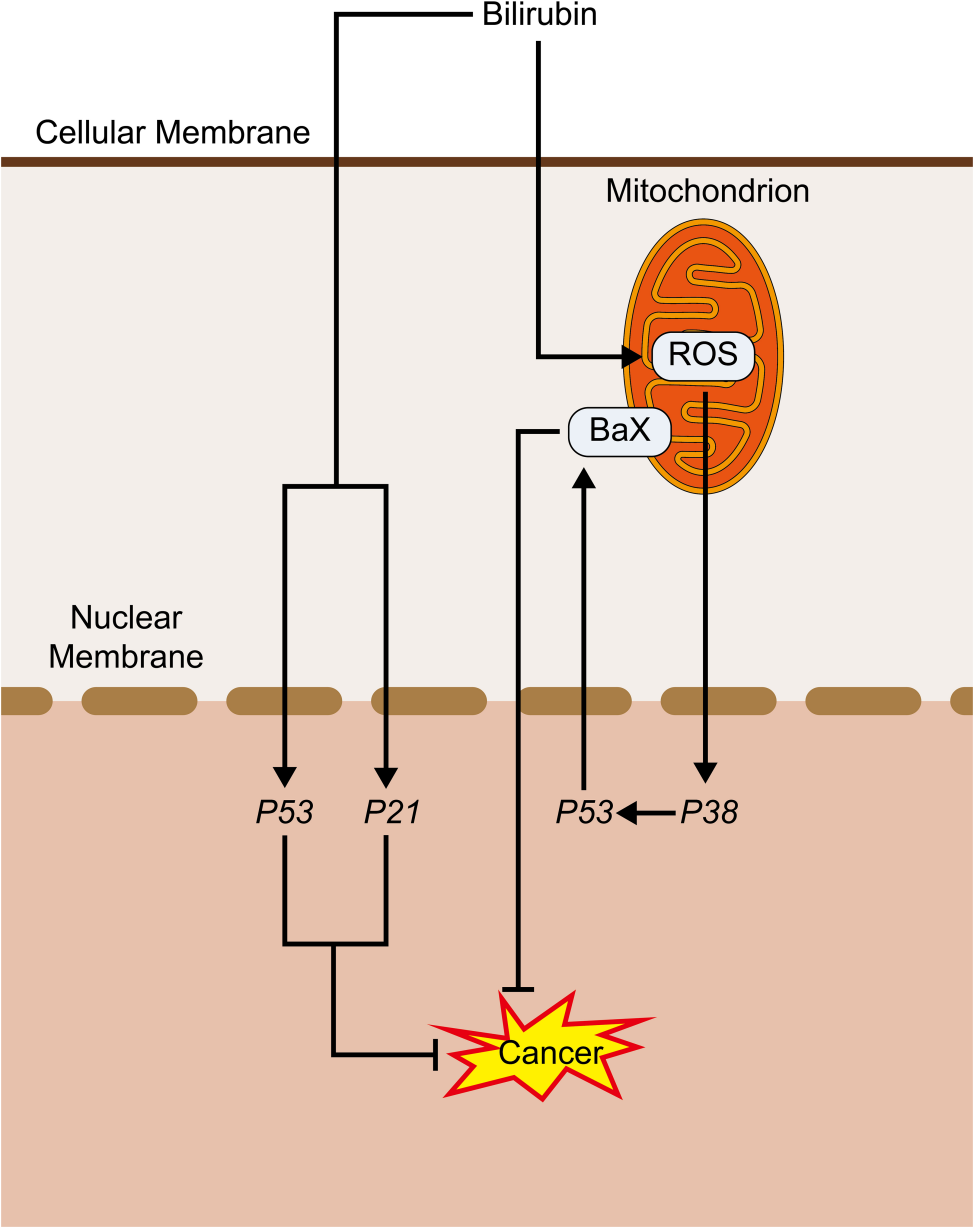
Bilirubin promotes cancer inhibition pathway: Bilirubin promotes ROS production in mitochondria, which activates *P38*, and its activated *P53* activates BaX, which promotes its pro-apoptotic process. Bilirubin can also directly activate *P53* and *P21* to play an anti-cancer role.

Bilirubin inhibits PKA-catalyzed histone phosphorylation, as well as cAMP binding to PKA regulatory subunits, through both competitive (109) and noncompetitive (110) mechanisms. PKA anchoring acts importantly in pseudopod formation and chemotactic cell migration (111). Activation of the cAMP/PKA pathway induces loss of stress fibers (112), activation of regular arrangement of collecting venules (Rac) and Cell Division Cycle 42 (Cdc42) (113, 114), formation of filamentous pseudopods and lamellar pseudopods (114, 115) microfilament assembly (116), and inhibition of the Rho family of GTPases activity (117, 118), events which happen at the tip of migrating cells. PKA also affects integrin-dependent migration in a variety of cells (119) and PKA and its phosphorylated substrates are enriched in cellular protrusions (111, 120). Several types of cancers utilize the cAMP/PKA signaling pathway to behave malignant features of cancer, such as invasion, relocation, adhesion, and proliferation. PKA has been proven to inhibit the expression of malignant features of cancer by inhibiting the cAMP/PKA signaling pathway. Such correlation has been demonstrated in ovarian cancer (121–123), glioblastoma (124), colorectal cancer (125), breast cancer (126) and pituitary tumors (127), esophageal squamous cell carcinoma (128), suggesting that bilirubin may inhibit tumor progression by suppressing PKA (**Figures 3**, **4**).

#### 5.1.3 Bilirubin inhibits tumors development by suppressing the immune escape of tumors

Bilirubin can not only have a promoting effect on the immune system, but also an inhibitory effect, and this inhibitory effect can have an inhibitory effect on tumors. Next, we will talk about the direct and indirect inhibitory influences of bilirubin on the immune system, respectively.

##### 5.1.3.1 Direct suppression of the immune micro-environment by bilirubin

Bilirubin acts as an inhibitor at many sites in the immune system, including immune cell proliferation, antibody secretion, and complement activation. Counterintuitively, while bilirubin can act as a suppressor of the immune system (129), serving to increase the rate of infection (130–132), this suppression can instead act as a cancer suppressor. As one study showed (133), unconjugated bilirubin disrupts the interaction between C1q and immunoglobulins, thereby suppressing the first step of the classical pathway of complement activation, which is activated by several factors that are cancer-promoting (134–136), so this inhibition of the classical pathway may act as a cancer-inhibitor. Studies carried out on jaundiced infants have shown (137, 138) that the lymphocytes extracted from them are in a state of proliferation inhibition and their total IgA and IgM levels are reduced. In terms of bilirubin’s effects on immune cells, a 2017 study showed (139) that unconjugated bilirubin upregulates CD39 and thus inhibits Th17 immunoreactivity, this promotes several cancers, such as breast, colorectal, and ovarian cancers (140–142), and thus, the inhibition of TH17 by unconjugated bilirubin can play a role in inhibiting related cancer effects. Other than this, UCB activates both extrinsic and intrinsic pathways of apoptosis as reflected by markers such as CD95, caspase-8, Bax, MMP, cytoplasmic Ca^2+^, caspase-3, and DNA fragmentation (143). This literature also points out that glutathione is a key molecule in preventing UCB-induced cell death, which may help to investigate the use of bilirubin in cancer control.

##### 5.1.3.2 Indirect suppression of the inflammation by bilirubin

Bilirubin can restrain the development of cancer by reducing inflammation and inhibiting oxidative stress. Inflammation is a pathological process featuring tissue damage or destruction resulting from a variety of chemical and cytological reactions. It usually has typical symptoms such as redness, swelling, fever, pain, and function loss. As mentioned in the previous section, ROS are pro-inflammatory, whereas bilirubin is known to scavenge ROS and reduce oxidative stress. In a 2020 study (144), Lee et al. treated acute colitis in a mouse model of inflammatory bowel disease using hyaluronic acid-bilirubin nanoparticles, which reinstated the epithelial barrier in the mice as well as modulating the intestinal microbiota, showing promising anti-inflammatory properties by increasing overall abundance and diversity. In another study done on a rat model of acute pancreatitis (145), Yao et al. used a similar approach also to find that bilirubin had an anti-inflammatory effect, and this treatment was demonstrated to protect alveolar cells from damage and alleviate acute pancreatitis. The anti-inflammatory effect of bilirubin can be side-stepped by the fact that serum bilirubin is negatively related to the markers of oxidative stress in serum (146, 147) and markers of inflammation such as C-reactive protein levels (148, 149). Many studies demonstrated that a variety of chronic inflammatory conditions are strongly associated with cancer development, such as IBD (150) and pancreatitis (151–153). This indicates that bilirubin has an anti-inflammation effect with a possible reduction in inflammation-associated cancers by this pathway.

##### 5.1.3.3 Bilirubin acts as a ligand that binds to cellular receptors and produces oncogenic effects

The effect of bilirubin on cancer has long been thought to be primarily associated with its strong antioxidant properties. However, in recent years, a growing amount of studies have shown that bilirubin acts as a ligand role in cellular signaling processes and through this role has an impact on cancer development.

Receptors that can bind bilirubin include aryl hydrocarbon receptor (AhR), peroxisome proliferator-activated receptors α (PPARα), constitutive androstane receptor (CAR), fatty acid-binding proteins (FABPs), etc., which will be described below.

AhR is a widespread receptor found in many organs, body tissues, and cell groups (154), exhibiting both anticancer and oncogenic properties (155). Experiments carried out on cells cultured *in vitro* have shown that both bilirubin and biliverdin are endogenous agonists of the AhR (97). *In vitro* experiments in human colon cancer cells, agonist stimulation of the AhR may mediate cancer cell cycle arrest (156). In contrast, bilirubin and biliverdin, as endogenous agonists of the AhR, may reduce the proliferation of cancer cells due to the AhR stimulation.

PPARs are multi-structural domain proteins belonging to the nuclear receptor superfamily, acting mainly as ligand-activated transcription factors (157). PPARα can upregulate genes involved in fatty acid transport, as well as the fatty acid β-oxidation process related to the mitochondria and peroxisomal, to facilitate the intake, utilization, and catabolism of fatty acid (13). Experimental studies carried out in rodents have also shown that bilirubin reduces liver fat accumulation by binding to PPARα (13, 158–160), and the mechanism behind this may be an increase in both number and function of mitochondria, leading to a reduction in lipid accumulation (161–163). Several studies in different human patient populations have also demonstrated that bilirubin levels in serum, especially direct bilirubin, are negatively correlated with the prevalence of nonalcoholic steatohepatitis (64, 164–167). Because bilirubin protects the liver by reducing the incidence of nonalcoholic steatohepatitis, which another study showed may progress to cirrhosis and HCC (168, 169), bilirubin may inhibit cancer by binding to PPARα.

**Figure 5:**
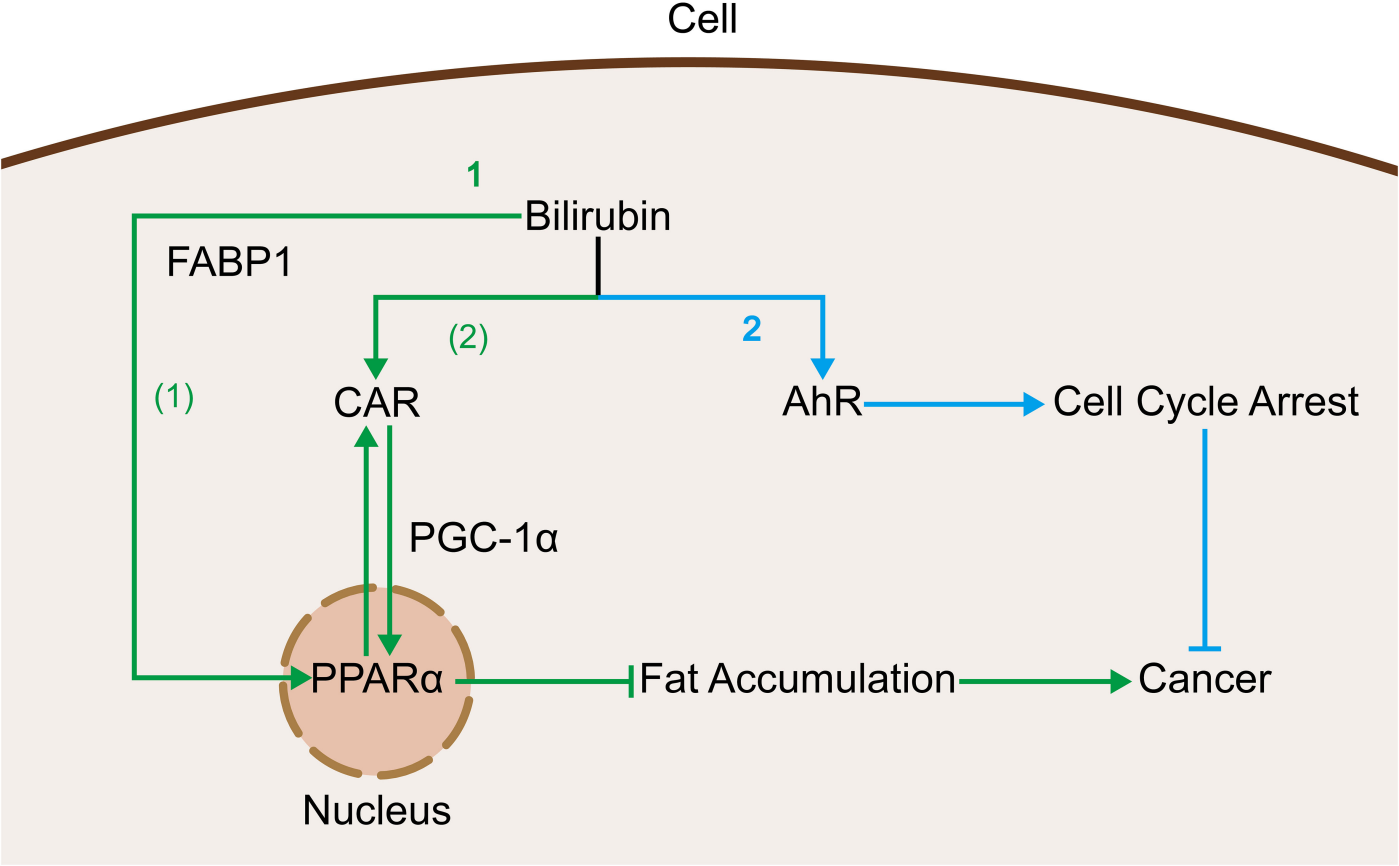
Bilirubin acts as a ligand that binds to cellular receptors and produces oncogenic effects: 1. Bilirubin activates PPARα via two pathways to inhibit fat accumulation and thus related cancers. (1) Bilirubin is delivered to the nucleus by binding to FABP1 thereby binding to PPARα. (2) Bilirubin inhibits fat accumulation by promoting CAR expression and thereby contributing to its crosstalk with PPARα. 2. Bilirubin binds to AhR and promotes cell cycle arrest thereby inhibiting cancer.

CAR and FABPs are two cellular receptors that are capable of binding to and exerting effects on bilirubin, and the mechanisms of action of both are related to PPARα as mentioned above. Firstly, CAR is introduced, belonging to the nuclear receptor superfamily (170), and is enriched in the liver (171). It has been shown that intracellular bilirubin has ligand transcription factors for CAR (172, 173), which may enhance its transcriptional levels. In contrast, CAR crosstalks with peroxisome proliferator-activated receptor (PPAR) (174), and this crosstalk may allow bilirubin to reduce the incidence of nonalcoholic steatohepatitis through the interaction between CAR and PPAR (175), which in turn reduces the incidence of HCC. Then there are FABPs, FABPs are a class of low molecular weight intracellular proteins that bind efficiently to bilirubin (176) and deliver it to the PPAR in the nucleus (177), and this delivering action contributes to bilirubin’s binding to and functioning on the PPAR. In contrast, bilirubin, which is a strong regulator of PPARα (13) and PPARγ (178), can also trigger the expression of FABP1 through the regulation of PPAR. In studies of cancers such as HCC (179, 180) and colorectal cancer (181, 182), it has also been found that a high incidence of cancer is correlated with low expression of FABP1, which might be associated with the bilirubin-FABP1 interactions described previously (**Figure 5**).

### 5.2 Tumor-promoting effects of bilirubin

When at high values in the normal range, bilirubin may inhibit cancer, but bilirubin may promote cancer under certain circumstances. For instance, a study in 2024 showed that when the concentration of bilirubin in serum reaches a certain value, the antioxidant effect of bilirubin begins to reduce, and even induces oxidative stress and thus promotes the development of cancer (183). In other studies, bilirubin has shown some promoting effects in cancers such as the cancers in the liver (18, 184) and lung (185). This indicates that the relationship between bilirubin levels and cancer risk is not simply linear and that both too low and too high concentrations may increase cancer risk (22, 186, 187). For the use of bilirubin in cancer therapy, research on the dual effects of bilirubin on cancer is a must, fewer studies have been conducted on its cancer-promoting mechanisms. The cancer-promoting effects of bilirubin may work through the following mechanisms.

#### 5.2.1 Bilirubin promotes tumor development by altering immune cell phenotype

Bilirubin can have a promoting effect on some sites of the immune system and thus on tumors. As shown in an earlier publication, unconjugated bilirubin stimulates microglia primary cultures to respond by acquiring a phagocytic phenotype, which is transformed into an inflammatory response featuring secretion of the pro-inflammatory cytokines interleukin (IL)-1β, IL-6, and tumor necrosis factor (TNF)-α, increased regulation of cyclooxygenase (COX)-2, and enhanced the activities of MMP- 9 and -2 (188). In another study, an ϵ-polylysine-bilirubin (PLL-BR) coupling was designed and synthesized to act as a method for bilirubin solubility and delivery. *In vitro* experiments, it enhanced M2-type macrophage polarization, resulting in elevated cytoprotective effects against antioxidant and inflammatory conditions; *in vivo* experiments in diabetic mice, compared to untreated islets, PLL-BR-coated islets led to the induction of an anti-inflammatory response featuring higher M2 macrophage markers levels and localized vascularization (189).

In the above description, cancer development has been associated with inflammation in several ways, and the narrative here suggests that bilirubin can also promote inflammation in several ways that may promote cancer. Whereas M2 macrophages can exert cancer-promoting effects in several ways, such as directly promoting cancer cell metastasis (190), promoting angiogenesis (191), and inhibiting tumor-killer cell activity (192), this suggests that bilirubin may promote cancer through this mechanism.

Although it has been shown that bilirubin is mainly inhibitory in its effect on the proliferation of tumors, it can also exhibit promotional properties in some aspects, and further studies on its inhibitory properties will help to generate new therapies for tumors, while studies on its promotional properties can give us a better knowledge of the two-faced nature of bilirubin, which can be used to aid in the side-by-side development of new treatments for tumors.

## 6 Therapeutic significance of bilirubin

As mentioned above, bilirubin is a substance with two sides: it is cytotoxic at high concentrations. Still, when bilirubin is only mildly elevated, such as in GS, bilirubin substantially benefits humans in maintaining good health and reducing the incidence of various oxidative stress-mediated diseases(including tumors). That is why there have been attempts to increase bilirubin levels in the body (mimicking the GS state) to prevent or treat cancer. Here are a few ways to increase the concentration of bilirubin in your body.

### 6.1 Dietary supplementation of bilirubin-like structure

Since bilirubin-like structures in plants share similar structure and function with bilirubin, complementing natural bilirubin-like structures through dietary supplementation is an effective way to elevate bilirubin levels, and one of the inexpensive and most studied bilirubin-like structures is Phycocyanobilin (PCB).PCB is a natural blue-coloring chromone with a linear tetrapyrrole structure in cyanobacteria and red algae and binds to phycocyanin (PC). It has antioxidant, anti-inflammatory, immunomodulatory, and anticancer activities due to its similarity to biliverdin or bilirubin (193). Currently, several animal experiments are proving the efficacy of PC in preventing or treating various diseases, as well as clinical trials of PC in cancer.

Spirulina, a cyanobacterium with various nutritional and therapeutic properties (194), is an important PC and allophycocyanin (APC) source in the phycobiliprotein family. There is research proving that the proliferation of pancreatic cancer cells was greatly inhibited *in vitro* by PC purified from Spirulina and that the inhibitory effect on the growth of a broad-spectrum cancer cell was enhanced with increasing doses of PC (195). Yang et al. found that the consumption of Spirulina by cancer patients during the first two cycles of chemotherapy increased IgM levels and CD8^+^T cell counts *in vivo*. This suggests that spirulina can reduce the incidence of myelosuppression and enhance immune function in tumor patients (196). Another study indicated that taking Spirulina reduced HBsAg levels in patients with chronic hepatitis B who were on continuous nucleoside analogs, and relieved liver inflammation, hepatic steatosis, and cirrhosis, thereby potentially lowering the probability of HCC (197). The above experiments show that PC has a widely suppressive effect on various tumor cells, which has crucial clinical significance.

Therefore, scientists’ research has become the direction of isolating and purifying PCB from natural algae to obtain a high concentration and maintain biological activity. Brião et al. produced food-grade PCs from spirulina by increasing the purity of PCs from 0.53 to 0.76 using phosphate buffer extraction combined with ultrafiltration and diafiltration (198). Furthermore, an investigator conducted ultrafiltration experiments using a 0.02g/mL Spirulina water extract sample. The purification/fractionation steps were carried out utilizing a polyethersulfone membrane. After the procedure, the purity of the rough extract improved from 0.74 to 0.93 due to the elimination of approximately 91.7% of the DNA, and the purification procedure was enhanced by applying six percolation cycles, forming the PC extract whose purity is up to 1.16, making it more favorable for food intake and biomedical applications (199).

In addition, it has been shown that the addition of 3 mM succinic acid to Spirulina medium increased biomass production rates to 164.05 mg/L(2-fold higher than the control)and increased PC production rates to 26.70 mg/L(3-fold higher than the control) (200). Moreover, the use of organic acids not only boosted the thermal stability of the PC but also increased the purity to 2.2.

It remains a challenge to develop a green and sustainable method to purify PCBs from algae to obtain high purity, suitable for biomedical applications.

### 6.2 Drug-induced GS status

**Figure 6:**
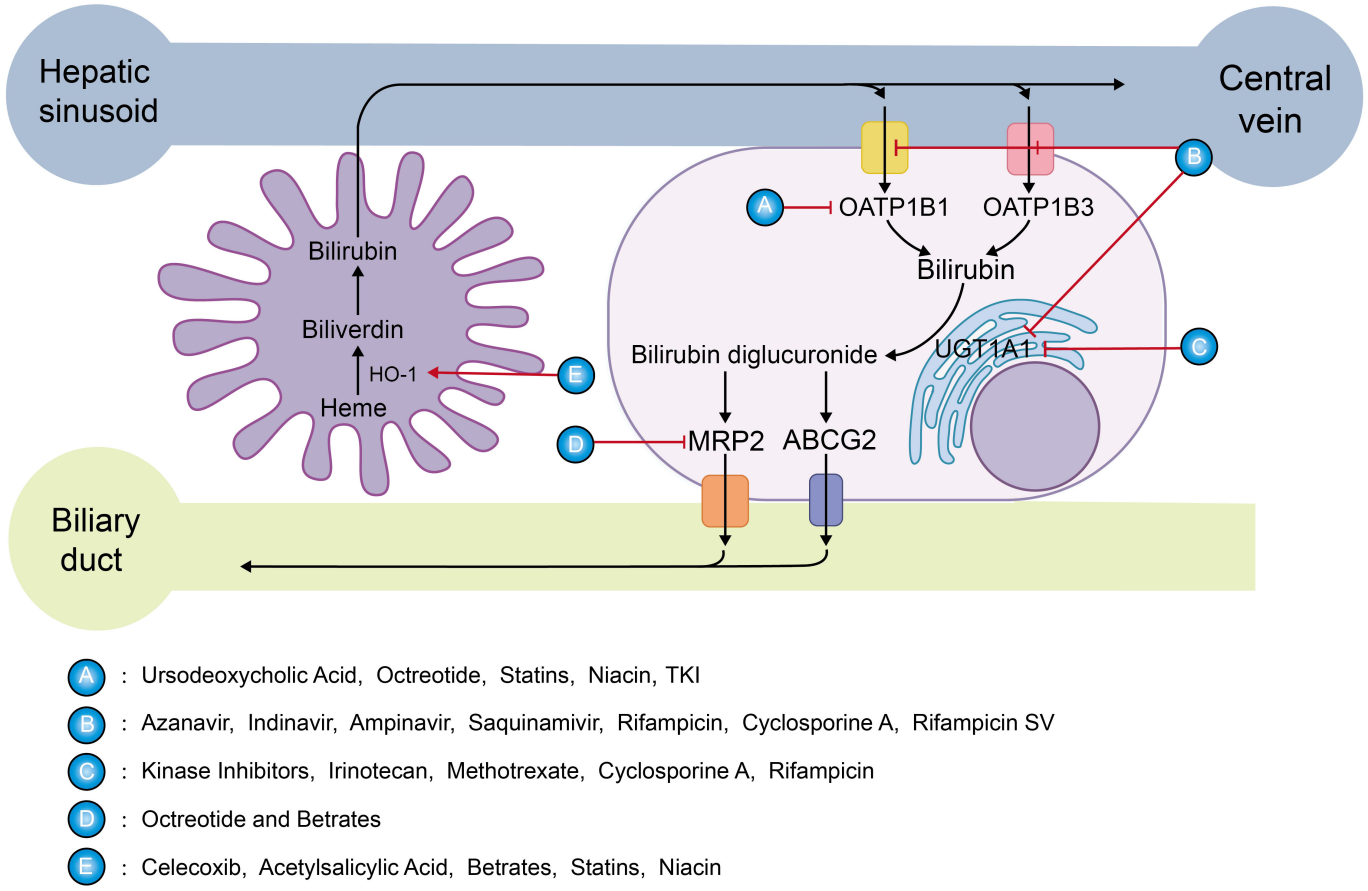
Targets of mildly elevated bilirubin concentrations induced by various drugs: The sharp red arrow suggests a promoting effect, while the blunt red one indicates inhibition. Class A drugs act by inhibiting OATP1B1, preventing the entry of UCB into the hepatocyte. Class B drugs inhibit OATP1B1, OATP1B3, and UGT1A1.Class C drugs inhibit UGT1A1 which affects the aldolization of bilirubin. Class D drugs inhibit bilirubin excretion through MRP2.Class E drugs induce HO-1 expression to promote bilirubin production.

Various drugs can affect key enzymes in bilirubin metabolism, resulting in mildly elevated serum bilirubin levels (mimicking a GS state) (**Figure 6**). This can potentially prevent and treat cancer to some extent. In addition, pharmacologic hyperbilirubinemia is typically benign and reversible, without underlying severe liver lesions such as cholestasis or hepatocyte necrosis. Therefore, drug induction can be considered a safe and reliable approach.

As is well known, ursodeoxycholic acid can hinder UCB from entering liver cells for transformation by inhibiting OATP1B1, leading to a notable increase in bilirubin concentration. A randomized controlled trial showed that low-dose ursodeoxycholic acid (500 mg) increased serum bilirubin concentrations by 30% (201). Furthermore, it has been reported that the use of ursodeoxycholic acid seems to have a positive influence on the prevention of radiation-induced liver disease after radioembolization in patients with liver metastases from breast cancer (202).

Atazanavir is an HIV protease inhibitor. A retrospective cohort study of 1,020 HIV patients demonstrated that HIV patients taking atazanavir had mildly increased serum bilirubin levels and a significantly decreased risk of developing new-onset cardiovascular disease (203). This effect appears to be reliably induced by indirect hyperbilirubinemia mediated by competitive inhibition of UGT1A1, which induces a Gilbert phenotype. In addition, atazanavir also inhibits OATP1B1 and OATP1B3, and indinavir, amprenavir, saquinavir, rifampicin, cyclosporin A, and rifamycin SV have the same mechanism (204, 205).

The suppression function of octreotide on OATP1B1-mediated transport is stronger, while it exhibits weaker inhibitory effects on OATP1B3 and MRP2, which is consistent with clinical observations. Hyperbilirubinemia occurs in a small portion of patients accepting octreotide treatment (206). If properly applied, it may inhibit cancer development by mimicking the GS state.

Clinically widely used NSAIDs, such as celecoxib (207) or acetylsalicylic acid (208), can induce HO-1. According to reports, after 24 hours of treatment with celecoxib, an increase in its concentration can enhance the levels of HO-1 mRNA and protein expression in endothelial cells of humans, and the bilirubin content reaches its highest level after 48 hours of celecoxib treatment.

A Mendelian randomization analysis study (209) detected that lowering LDL-C and PCSK9 protein levels by PCSK9 variants was relevant to an increase in CB, demonstrating that direct bilirubin is increased with the use of newer lipid-lowering therapies like the preprotein convertase chytokeratase/kexin type 9 (PCSK9), monoclonal antibody inhibitors: alirocumab and evolocumab, and hepatic PCSK9 siRNA inhibitor inclisiran. PCSK9 is inversely related to liver function and bilirubin (210, 211). Similarly, the drugs of the fibrates (212, 213) upregulate the expression of HO-1 mRNA and protein to increase bilirubin levels. Whereas statins (214–216), and niacin (217) in addition to the HO-1 induction mentioned above, include inhibition of basolateral transport of unconjugated bilirubin, the latter may be more important.

Kinase inhibitors such as erlotinib, nilotinib, pazopanib, regorafenib, sorafenib, and verofenib inhibit UGT1A1. Since the clinical steady-state C_max_ concentration is higher than their IC50, these TKI agents can make patients with a high incidence of hyperbilirubinemia by effectively inhibiting UGT1A1 (218, 219). In addition, it has been shown that most of the FDA-approved tyrosine kinases (TKIs) dramatically inhibit OATP1B1 for its function.

Irinotecan (220), methotrexate (221), cyclosporine A (222), and rifampicin (204) share similar UGT1A1 inhibitory activity in the treatment of cancer.

It is worth noting that hyperbilirubinemia caused by a variety of drugs is often seen as a symbol of deferred drug biotransformation and signs of drug-induced hepatic injury, so care should be taken to control the bilirubin concentration during the clinical application of drugs to induce a GS state; excessively high levels may be counterproductive. Bilirubin concentration levels and drug side effects should still be monitored several times while using these drugs.

### 6.3 Targeted bilirubin nanoparticles

Tumors generate large amounts of ROS during rapid metabolic proliferation, which can promote tumor cell survival, invasion, proliferation, and metastasis by regulating various signaling pathways (223, 224). Therefore, bilirubin can exert anticancer effects by scavenging reactive oxygen radicals in cancer cells. Bilirubin nanoparticles (BRNPs) can preferentially accumulate in tumors due to enhanced permeability and retention (EPR) effects (225). In addition, bilirubin’s rapid ROS and light response, efficient photothermal conversion, biodegradability, and biocompatibility make it more suitable than other liposomes as a carrier for encapsulating anticancer drugs (**Table 1**).

**Table 1:** Bilirubin-targeting nanomedicines for cancer treatment.

Classifications	Nanomedicines	Loaded drugs	Experimental models	Therapeutic effects	Tumor Types	Refs
As a multi-stimulus reactant	DOX@BRNPs	DOX	Xenograft mice bearing A549 tumors	Tumor growth was inhibited by 55.0% and up to 71.9% when combined with laser irradiation	Lung adenocarcinoma	(226)
	TH-302@BR- Chitosan NPs	TH-302	HeLa tumor-bearing mice.	Drug cytotoxicity was significantly increased and tumor hypoxia was enhanced	Cervical cancer	(227)
	SC144@HABN	SC144	MC38 tumor-bearing mice, BALB/c mice bearing 4T1 tumors	Combined anti-PD-L1 therapy significantly inhibits tumor growth in both mouse models	Colon carcinoma, breast cancer	(228)
	DOX@HABN	DOX	HeLa tumor-bearing mice.	Inhibits tumor growth by approximately 83.8%	Cervical cancer	(229)
	Ce6/BR-FFVLK-PEG	PTX, IND	4T1 breast cancer BALB/c nude mice models	Combined laser therapy achieved an inhibition rate of 85.27% ± 12.80% and significantly inhibited lung metastasis.	Breast cancer	(230)
As a photosensitizer for diagnosis and treatment	CisPt@BRNPs	Cisplatin	HT-29 human colorectal cancer xenograft tumor model	Induced over 70% of tumor cell death and achieved tumor visualization	Colon carcinoma	(231)
	PEG-BR/CWONPs	CaWO4	Murine xenograft models of head and neck cancer	Combined X-ray irradiation enhances the efficacy of anti-cancer treatment	Head and neck cancer	(232)
	Mn@bt-BRNPs	DOX	Xenograft mice carrying human lung adenocarcinoma cells (A549)	Effectively inhibits the growth of A549 lung cancer cells and induces T1-weighted MRI signal enhancement	Lung adenocarcinoma	(233)
	Self-assembling endogenous biliverdin	–	MCF-7 xenograft tumor model	Induces efficient photothermal ablation of tumors under near-infrared irradiation, better imaging of tumors in ultrasound and MRI	Breast cancer	(234)

DOX, doxorubicin; @, loaded with; BR, bilirubin; NPs, nanoparticles; TH-302, evofosfamide; SC144, a gp130 inhibitor; HA, hyaluronic acid; BN, bilirubin nanoparticles; Ce6, chlorin e6; FFVLK, Phe-Phe-Val-Leu-Lys peptide; PEG, polyethylene glycol; PTX, paclitaxel; IND, Indoximod; cisPt, cisplatin; CWO, CaWO4; Mn, manganese ion; bt, biotinylate; MRI, magnetic resonance imaging.

#### 6.3.1 Bilirubin as a multi stimulus reactant

Bilirubin is insoluble in water because of the formation of intramolecular hydrogen bonds by its hydrophilic groups (carboxylic acid and amide bonds). However, bilirubin can target ROS and be oxidized to biliverdin, resulting in increased water solubility. When irradiated with light of a suitable wavelength, bilirubin can also occur in photoisomerization (through intramolecular hydrogen bonds breaking) and be converted to the more water-soluble photoisomer, which is readily disposed of and eliminated by the liver and kidneys (226, 235). This ability to respond to both ROS and external light stimuli demonstrates that bilirubin can be used as a multi-stimulus response system to construct nanomedicines.

In 2016, Lee et al. first developed BRNPs for cancer therapy (226). They combined bilirubin and polyethylene glycol into a coupling via a steady amide bond and loaded the anticancer drug doxorubicin (DOX) into BRNPs via film-forming and rehydration to form DOX-loaded BRNPs (DOX@BRNPs). *In vivo*, experiments in xenograft mice bearing human lung adenocarcinoma cells indicated that tumor growth was restrained by 27.8% in mice treated with free DOX, 55.0% in the DOX@BRNPs group, and even as high as 71.9% in the laser-irradiated DOX@BRNPs group. This indicates that the use of BRNPs can greatly enhance the anti-tumor efficacy of DOX chemotherapy drugs. In addition, 38.1% of tumor growth was inhibited when treated with BRNPs individually, suggesting that BRNPs, as well as free bilirubins, also have inherent anticancer efficacy.

Another interesting experiment was published (227). The bilirubin-chitosan conjugate (BR-Chitosan) was labeled with Sulfo-Cyanine7 fluorescent dye and injected into HeLa tumor-bearing mice. Eight hours later, it primarily accumulated in the tumor region, whereas free Sulfo-Cyanine7 exhibited a systemic distribution. Experimental results indicate that BR-Chitosan exhibits significant tumor-targeting properties. In an exception to the catabolism of the BR-Chitosan at the tumor site to release the internal hypoxia-activated prodrug HAPTH-302, it also exhibited intrinsic oxygen-consuming properties in the tumor region in the presence of overexpression of ROS, including H_2_O_2_, which greatly increased the degree of hypoxia around the tumor tissues and converted HAPTH-302 from nontoxic to toxic in a hypoxic environment, exerting selective antitumor effects. Bilirubin not only compensates for the limitation of HAPTH-302’s inability to function in the region of normoxic tumors but also has a strong absorbance that allows for chemo-thermal synergistic treatment. It also has a simpler and safer composition than other deoxidizers and carriers. In HeLa tumor-bearing mice, drug cytotoxicity was significantly increased and tumor hypoxia was enhanced.

To enhance the antitumor effects, Lee et al. sought to further refine BRNPs by incorporating immune responses. Hyaluronic acid (HA) has targeted CD44 and immunomodulatory properties. Recently, they developed a hyaluronic acid-bilirubin nanoparticles loaded with SC144(SC144@HABN) (228). Experimental data showed that intravenously injected HABN accumulated in tumor cells and tumor-associated myeloid cells, (possibly as a result of the enhanced permeability and retention effect and targeting of CD44, ROS effect) allowing SC144 to act: radicalizing macrophages into the M1 phenotype, inducing killing effect of CD8^+^ T cells, enhanced PD-L1 expression in tumor cells and inducing immunogenic cell death *in vitro*. The significant tumor-targeting and lysis-releasing effects of HABN vectors attenuated the toxicity of SC144 on human normal cells and CD8^+^ T cells, and enhanced the synergistic anti-cancer efficacy of immunotherapy. In addition, Animal experiments have shown that the SC144@HABN+anti-PD-L1 combination treatment can complementarily increase anti-neoplastic efficiency and effectively eliminate MC38 colon tumors and immune checkpoint blockade-resistant 4T1 breast tumors. Subsequently, articles have also reported that HABN containing adriamycin (DOX@HABN) showed significant tumor targeting and synergistic anti-tumor capabilities in HeLa-loaded mice (229).

In addition, a Ce6/BR-FFVLK-PEG nanomedicine encapsulating dimeric paclitaxel (PTX) & indolimod (IND) was reported (230) Its surface-covered macrophage membrane facilitates nanomedicine circulation and tumor targeting. Chlorin e6 (Ce6) or BR can generate ROS under laser beam irradiation, which triggers the conversion of micelles into nanofibers and facilitates the release of core anticancer agents. Growth and lung metastasis of breast cancer *in situ* were considerably inhibited by this nanomedicine through BR-induced photodynamic therapy, PTX-induced typical immunogenic cell death (ICD) effect, and activation of immune response in combination with IND.

#### 6.3.2 Bilirubin as a photosensitizer for diagnosis and treatment

Due to bilirubin’s high photosensitivity (PS), blue light is often utilized to reduce unconjugated bilirubin levels in neonates or preterm infants. When laser light irradiates accumulated PS reagents (e.g., bilirubin), photoactivation generates toxic ROS and induces cell death to provide local antitumor therapy. Moreover, the liganding of bilirubin with particular metal ions may enhance its effectiveness in photoacoustic imaging (PAI) and photothermal therapy (PTT).

Inspired by the binding mode of melanin gallstones, Lee et al. exploited cisplatin-chelated bilirubin nanoparticles (cisPt@BRNPs) (231). They found that under laser irradiation, with its great photothermal conversion efficiency, it could generate considerable heat, causing cell death in more than 70% of HT-29 human colorectal adenocarcinoma cells (while cisplatin exerted a small anti-tumor effect). Interestingly, substantial photoacoustic activity was generated by cisPt@BRNPs, enabling tumor visualization, which can be used as a PAI probe for cancer diagnosis and treatment.

Because of the shallow depth of light penetration in the tissue, it cannot treat tumors deeper in. So novel PEGylated bilirubin-encapsulated CaWO4 nanoparticles(PEG-BR/CWO NPs) were reported by Pizzuti et al (232). When the nanoparticles were irradiated by X-rays, the CaWO4 core emitted ultraviolet and blue light, which was absorbed by bilirubin to produce a photodynamic effect, generating ROS to enhance cancer cell death. Animal studies of head and neck cancer transplants have shown that combining radiation and photodynamic therapy enhances the anti-cancer treatment effect.

Recently, Mn^2+^-chelated, biotinylated bilirubin nanoparticles (Mn@bt-BRNPs) were reported. Unlike conventional MR contrast agents, BRNPs release internal Mn^2+^ in response to ROS stimulation, which allows for better display of ROS in the tumor and tumor microenvironment, thereby inducing T1-weighted MRI signal enhancement. Moreover, experimental data indicated that adriamycin-loaded Mn@bt-BRNPs could effectively inhibit the growth of A549 lung cancer cells (233). This combined antitumor effect and tumor visualization in one of the BRNPs is a significant advantage of them as nanocarriers. Similar bilirubin nanomedicines have been reported to monitor the progression of cirrhosis (236).

In addition, BV nanoparticles with long-term fixity were constructed by Xing et al. through supramolecular self-assembly of BV and metal-binding short peptides. Metal ions, being incorporated into the BV nanoparticles such as Mn^2+^, can strengthen their photostability and endow them with magnetic resonance. Data from *in vivo* studies show that its selective accumulation in tumors can locally raise the temperature of tumors under near-infrared illumination, thus leading to the induction of effective photothermal tumor ablation for selective treatment (234). In addition, BV nanoparticles can also be used as multimodal contrast agents for better imaging of tumors in ultrasound and magnetic resonance, thus having great potential in precision medicine.

It is worth noting that the anticancer and immunomodulatory role of bilirubin in nanomedicines was overlooked in some of the above experiments, which focused on its high photothermal conversion efficiency as a photosensitizer on tumors, without noting that the anticancer role of bilirubin/biliverdin as a ROS scavenger in its own right should not be underestimated. Moreover, BRNPs have better efficacy in ROS-overproducing tumor cells than in ROS-low-expressing tumors. The therapeutic efficacy of passively targeted BRNPs dependent on the EPR effect is largely influenced by differences in the strength of the EPR effect exhibited within different tumors (225). It remains a challenge to effectively utilize the benefits of bilirubin to target tumor cells highly selectively.

## 7 Conclusions and outlook

Bilirubin has a complex interaction with cancer, and bilirubin can inhibit tumorigenesis and development through a variety of mechanisms, including influencing cellular redox reactions, signaling, and the immune system, while the effects of cancer on bilirubin have derived several prognostic models. Current research on the effects of bilirubin on tumors has some contradictory results and perhaps unexplored underlying mechanisms. Some studies have found antiproliferative effects of bilirubin in specific human tissue cells, but have not gone on to investigate its role in the corresponding tumors. The effect of bilirubin on the intestinal flora, which plays a key role in its metabolism, has also not been explored.

Dietary supplementation with PC and drug induction can elevate serum bilirubin concentrations, but only modest elevations are strongly associated with reduced cancer incidence. In constructing BRNPs, bilirubin is a versatile carrier for antitumor drugs and a photosensitizer for diagnosing and treating cancer, enabling better imaging of tumors. However, the antitumor effects of bilirubin itself were overlooked in most experiments. The efficacy of BRNPs in ROS overproducing tumors was better than that in ROS low-expressing tumors, which might be the limitation of BRNPs. Despite this, bilirubin-targeted nanomedicines remain a suitable approach for future cancer prevention and treatment.
